# Human vault RNA1-1, but not vault RNA2-1, modulates synaptogenesis

**DOI:** 10.1080/19420889.2021.1909229

**Published:** 2021-04-14

**Authors:** Shuji Wakatsuki, Moeka Ohno, Toshiyuki Araki

**Affiliations:** Department of Peripheral Nervous System Research, National Institute of Neuroscience, National Center of Neurology and Psychiatry, Kodaira, Tokyo, Japan

**Keywords:** Synaptogenesis, vault, non-coding RNA, mapk

## Abstract

The small non-coding vault RNA (vtRNA) is a component of the vault complex, a ribonucleoprotein complex found in most eukaryotes. vtRNAs regulate a variety of cellular functions when unassociated with the vault complex. Human has four vtRNA paralogs (hvtRNA1-1, hvtRNA1-2, hvtRNA1-3, hvtRNA2-1), which are highly similar and differ only slightly in primary and secondary structure. Despite the increasing research on vtRNAs, a feature that distinguishes one hvtRNA from the others has not been recognized. Recently, we demonstrated that murine vtRNA (mvtRNA) promotes synapse formation by modulating the MAPK signaling pathway. Here we showed that expression ofhvtRNA1-1, but not hvtRNA2-1 increases the expression of synaptic marker proteins, ERK phosphorylation and the number of PSD95 and Synapsin I double positive puncta to an extent similar to that of mvtRNA, suggesting that hvtRNA1-1 may enhance synapse formation. This finding opens new perspectives to uncover the function of the different vtRNA paralogs.

The establishment of axon/dendrite polarity is a critical step in neuronal differentiation [1,2]. Neurodevelopmental disorders, including autism spectrum disorders, are characterized at cellular levels by abnormal establishment of neuronal connectivity during development [3,4]. Subcellular signaling, involving protein kinases, plays a significant role in the establishment and regulation of neuronal connectivity at synapses. Among such kinases, the mitogen-activated protein kinase (MAPK) signaling pathway leading to the activation of extracellular signal-regulated kinases-1 and −2 (ERK1 and ERK2) plays a key role in regulating local protein synthesis in dendrites, formation and stabilization of dendritic spines, and synaptic plasticity in the brain [5,6]. Unfortunately, the precise molecular mechanism for these regulations remains elusive

Small non-coding vault RNAs (vtRNAs) have been described as a component of the vault complex, a hollow-and-barrel-shaped ribonucleoprotein complex found in most eukaryotes [7,8]. It has been suggested that the function of vtRNAs might not be limited to simply maintaining the structure of the vault complex [9–11]. Recently, we revealed a novel role for vtRNA in synaptogenesis [12]. Using a model of synaptogenesis *in vitro*, we demonstrated that mvtRNA up-regulates synaptogenesis by activating the MAPK signaling pathway. mvtRNA binds to and activates mitogen activated protein kinase 1 (MEK1), and thereby enhances MEK1-mediated ERK activation. These results identified the regulatory mechanism of MAPK signaling pathway through mvtRNA as a novel molecular basis for synaptogenesis. Interestingly, a human vtRNA hvtRNA2-1 did not exhibit any effect on MEK1 kinase activity in an *in vitro* kinase assay. Human has four vtRNA paralogs [13]. To examine the possibility that other hvtRNAs might up-regulate synaptogenesis by amplifying MAPK signaling, we assessed the effect of expression of hvtRNA1-1 on synapse formation using the *in vitro* model (Figure 1). We found that expression of hvtRNA1-1, but not hvtRNA2-1, using lentivirus vectors in cultured cortical neurons increases the expression of synaptic marker proteins, ERK phosphorylation and the number of PSD95 and Synapsin I double positive puncta to an extent similar to that of mvtRNA. These results indicated that hvtRNA1-1, but not hvtRNA2-1, may up-regulate synaptogenesis by activating the MAPK signaling pathway.

**Figure 1.**
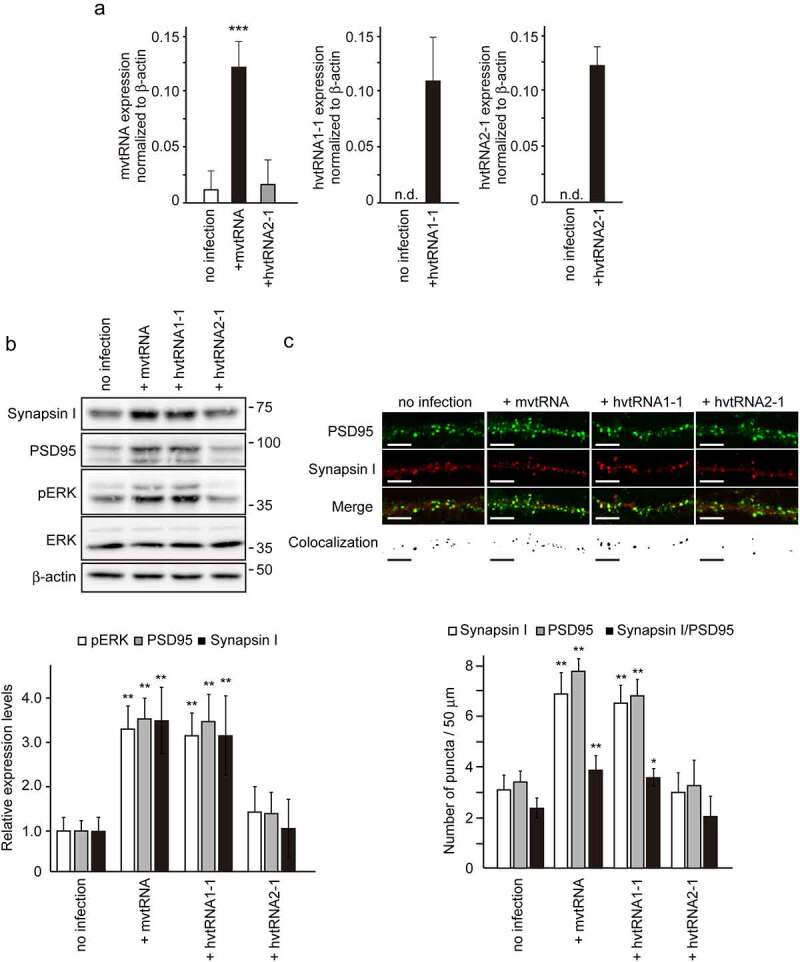
The small non-coding hvtRNA1–1, but not hvtRNA2-1, enhances synapse formation by the activation of ERK signaling

Overexpression of hvtRNA1–1 enhances synapse formation by the activation of ERK signaling. (**A**) Total RNA was prepared from cultured cortical neurons infected with lentivirus expressing mvtRNA, hvtRNA1–1, and hvtRNA2–1 and expression of each gene was analyzed by qPCR. Expression levels of each gene normalized to β-actin (mean ± SEM, n = 3) are shown. (**B**) Lysates were prepared from cultured cortical neurons infected with lentivirus expressing mvtRNA, hvtRNA1–1, and hvtRNA2–1. Representative immunoblots for the expression of the indicated molecules are shown. β-actin serves as a loading control. Phosphorylation levels of ERK (calculated as pERK/total ERK) and expression levels of Synapsin I or PSD95 normalized to β-actin relative to the control (no infection) (mean ± SEM, n = 5) are shown. (**C**) Synapse formation was assessed by double immunostaining with antibodies against PSD95 and Synapsin I. Representative photomicrographs of cultured cortical neurons at 14–16 DIV, and the colocalization panels show the double positive puncta extracted from merged images that were identified as synapses (shown in black). The number of Synapsin I-positive, PSD95-positive, and double positive synapses per dendrite length were shown. Scale bar, 25 µm. Significant differences from the control (*, *P* < 0.05; **, *P* < 0.01; ***, *P* < 0.001) were determined by a one-way ANOVA with Tukey’s post-hoc test. n.d, not detected.

Recent reports have suggested that vtRNA may function to regulate cellular patho-physiology [9–11]. In current paper, we demonstrated that hvtRNA1-1, but not hvtRNA2-1, enhances synapse formation by modulating the MAPK signaling pathway. Amort *et al*. also reported that hvtRNA1-1, but not other hvtRNA paralogs, has a protective effect against apoptosis in a cellular model of Epstein-Barr virus infection [14]. The sequence alignment of vtRNAs reveals two blocks of identical sequence for Pol III transcription [8,9]. The sequence homology of vtRNA2-1 with other three vtRNAs is limited mostly around these blocks. Thus, vtRNA2-1 is divergent in sequence from vtRNAs that are conserved among them. Bracher et al. demonstrated that the MAPK pathway is misregulated upon hvtRNA1-1 loss, thereby leading to induce apoptosis in human cancer cells [15]. They also found that a short stretch within its central domain is essential for conferring apoptosis resistance although the precise mode of molecular interaction between MAPK pathway and hvtRNA1-1 is unknown. Mutagenesis experiments and high-resolution structural analyses are expected to highlight the precise mode of interaction between MEK1 and vtRNA, with implications for the question of the binding specificity and the mechanistic model. Thus, together with previous observations, our current finding open new perspectives to uncover the function of the different vtRNA paralogs.

## Materials and methods

### Animals

All animals were maintained in accordance with the guidelines of the National Center for Neurology and Psychiatry. The technical protocols for animal experiments in this study were approved by a review committee for Animal Resources in the National Center for Neurology and Psychiatry.

### Antibodies

The antibodies used and their sources are as follows: mouse anti-β-actin antibody (622,101, BioLegend); rabbit anti-ERK antibody (4695, Cell Signaling); rabbit anti-phospho-ERK antibody (4377, Cell Signaling); mouse anti-PSD95 antibody (MA1-046, Thermo Scientific); guinea pig anti-PSD95 antiserum (124 014, Synaptic Systems); mouse anti-synapsin I antibody (MAB355, Millipore). Horseradish peroxidase (HRP)-conjugated (Vector Laboratories), Alexa565-conjugated, and Alexa488-conjugated (Molecular Probes) antibodies were used as secondary antibodies for detection.

### Cortical neuron culture

Cerebral hemispheres were removed separately from embryonic day (E) 14–16 C56BL/6J mice. Cells were dissociated with papain, and seeded at a density of 4 × 10^5^ cells/well onto 24-well plates coated with poly-L-lysine (Merck) and laminin (Merck) in DMEM containing 10% fetal bovine serum. From the third-day *in vitro*, the cells were maintained in Neuro-medium (Miltenyi Biotec) containing 2% Neuro-Brew-21 (Miltenyi Biotec) and 1 mM GlutaMAX (Thermo Scientific). The cells were cultured for 14–16 days, and then used for the immunoblot or immunostaining experiments.

### Immunoblot

For immunoblot analysis, cultured cells were homogenized in RIPA buffer (1% Triton X-100, 0.5% sodium deoxycholate, 0.1% SDS, 150 mM NaCl, 50 mM Tris–HCl, pH 7.5) containing phosphatase- (Cat. No. 07574–61, Nacalai tesque) and protease inhibitor cocktails (Cat. No. 25,955–11, Nacalai tesque). Equal amounts of protein were separated by SDS-PAGE, followed by immunoblotting. Immunoreactivity was visualized by using HRP-conjugated secondary antibodies and a chemiluminescent substrate (Wako Chemical). The chemiluminescent images were captured by LAS4000-mini and quantified using ImageJ software. Scans at multiple exposures were obtained to ensure that the results fell within the linear range of the instrument.

### Viral vectors and infection

mvtRNA, hvtRNA1–1, and hvtRNA2–1 were amplified by PCR using LA-taq from mouse brain or HEK293T cell cDNA library, and cloned into pLKO.1 lentivirus vector that contains a human U6 RNA polymerase III promoter. Lentiviral packaging was performed using HEK293FT cells, as previously described [16].

### Microscopy image acquisition

In the immunocytochemical analysis of cultured cortical neurons, cells were fixed with 4% paraformaldehyde in PBS, and then permeabilized with 0.2% Triton X-100 in PBS. An incubation with primary antibody was performed at 4°C overnight, followed by secondary antibody at room temperature for 1 h. Specimens were mounted using Vectashield mounting medium (Vector Laboratories). Immunofluorescence was observed under an inverted microscope (DMI 6000B; Leica) and analyzed using LAS AF software (version 3.2; Leica). Images were taken with a constant exposure time between all the conditions of the same experiment and were processed using Photoshop software (Adobe).

For synaptogenesis experiments, 14–16-day cultures were immunostained using antibodies against Synapsin I and PSD95. The number of double positive puncta per 50 µm-length of dendrites expressing the indicated molecules (n = 5 for each condition) were counted for quantification in each sample.

### Quantitative reverse transcription-polymerase chain reaction (RT-PCR)

Total RNA was extracted from cultured cortical neurons using the RNeasy MiniKit (Qiagen). Real-time quantitative RT-PCR was performed as previously described using the Applied Biosystems Prism model 7300 sequence detection instrument and a standard SYBR green detection protocol. The sequences of the PCR primers using the SYBR green method are as follows:

β-actin forward, 5ʹ-CCCCCAATGTATCCGTTGTG-3ʹ;

β-actin reverse, 5ʹ-CCAGTTGGTAACAATGCCATGT-3ʹ;

hvtRNA1-1 forward, 5ʹ-TTAGCTCAGCGGTTACTTCGACAGTTC-3ʹ;

hvtRNA1-1 reverse, 5ʹ-AAAAGGACTGGAGAGCGCCC-3ʹ;

hvtRNA2-1 forward, 5ʹ-GGGTCGGAGTTAGCTCAAGC-3ʹ;

hvtRNA2-1 reverse, 5ʹ-AAAGGGTCAGTAAGCACCCG-3ʹ;

mvtRNA forward, 5ʹ-CAGCTTTAGCTCAGCGGTTAC-3ʹ;

mvtRNA reverse, 5ʹ-AAGGGCCAGGGAGCGCCCGC-3ʹ.

The samples were run in duplicate. The relative amount of cDNA was determined in duplicate and calculated according to the 2^−ΔΔCT^method. The expression level for each gene of interest was normalized to β-actin.

## Statistical analysis

Results were expressed as the mean ± SEM. Differences between groups were examined for significance using a one-way ANOVA with Tukey’s post hoc test.
